# Precision Prescribing in the ICU: Evaluating BioFire® FilmArray® Pneumonia Panel and Standard Culture-Guided Therapy for Ventilator-Associated Pneumonia

**DOI:** 10.7759/cureus.111613

**Published:** 2026-06-27

**Authors:** Ravindra S Bohra, Sushant Khanduri, Sonu Sama, Barnali Kakati, Rakhee Sodhi Khanduri, Varuna Jethani

**Affiliations:** 1 Respiratory Medicine, Himalayan Institute of Medical Sciences, Swami Rama Himalayan University, Dehradun, IND; 2 Critical Care Medicine, HImalayan Institute of Medical Sciences, Swami Rama Himalayan University, Dehradun, IND; 3 Microbiology, All India Institute of Medical Sciences Rishikesh, Rishikesh, IND

**Keywords:** biofire, biofire filmarray pneumonia panel, culture sensitivity, icu outcomes, multiplex pcr, rapid diagnostics, ventilator-associated pneumonia

## Abstract

Background: Ventilator-associated pneumonia (VAP) is a leading cause of morbidity and mortality in ICUs. Timely identification of pathogens is critical to initiating targeted antimicrobial therapy. This study evaluated the clinical and diagnostic impact of the BioFire® FilmArray® Pneumonia Panel (BFPP; BioFire Diagnostics, LLC, Salt Lake City, UT, USA), a rapid multiplex PCR-based test, compared to conventional culture and sensitivity testing in patients with VAP.

Methods: A prospective observational longitudinal study was conducted in a North Indian tertiary care medical college. A total of 106 patients with VAP, diagnosed based on the National Healthcare Safety Network (NHSN) infection-related ventilator-associated complication (IVAC) criteria, were equally divided into two groups. Group A underwent both BFPP and culture testing; Group B was assessed using culture-based diagnostics alone. Demographic data, Acute Physiology and Chronic Health Evaluation II (APACHE II) score, ICU stay, ventilation duration, extubation timing, antibiotic usage, and 28-day mortality were recorded and analyzed.

Results: The mean APACHE II score was higher in the culture group (30.7 ± 6.2) than in the BFPP group (29.5 ± 7.1; p = 0.20), but was not statistically significant. ICU stay was significantly shorter in BFPP-positive patients (median nine days) than in culture-positive patients (median 13 days; p = 0.021). Early extubation within seven days was more frequent in the BFPP group (73.7%) compared to the culture group (52.9%; p = 0.047). While overall 28-day mortality was lower in the BFPP group (55.8%) compared to the culture group (62.3%), the difference was not statistically significant (p = 0.21). However, sex-stratified analysis showed significantly lower mortality among both males (p = 0.037) and females (p = 0.048) in the BFPP group. A concerning finding was the higher mortality rate in BFPP-negative patients (43.5%) compared to culture-negative cases (38.5%; p = 0.042).

Conclusion: The integration of the BFPP into the diagnostic workflow for VAP demonstrated improved intermediate clinical outcomes, including reduced ICU stay, earlier extubation, and shortened ventilation duration. While overall mortality benefits were not statistically significant, subgroup analyses indicated a potential survival advantage with BFPP-guided management, particularly in pathogen-positive cases. However, caution is warranted in interpreting negative molecular results without clinical correlation, as these may lead to delayed or inappropriate therapy. This study supports the adoption of molecular diagnostics as a complementary tool to conventional cultures in managing VAP, especially when paired with robust clinical judgment and antimicrobial stewardship.

## Introduction

Ventilator-associated pneumonia (VAP) is among the most frequent and serious infections in critically ill patients, particularly those admitted to ICUs [1]. Defined as pneumonia developing more than 48 hours after initiation of mechanical ventilation, typically via endotracheal intubation or tracheostomy, VAP is associated with substantial morbidity, mortality, prolonged ICU stay, and increased healthcare costs [2]. Risk factors include prolonged ventilation, advanced age, comorbid illnesses, and the presence of artificial airways, which bypass normal host defenses and facilitate microbial colonization [3].

The diagnosis of VAP remains challenging. Clinical features such as fever, purulent secretions, hypoxemia, and radiographic infiltrates are nonspecific and may mimic other pulmonary conditions [4]. Conventional culture sensitivity testing is the standard diagnostic approach, but its limitations are well recognized. Culture-based methods require 48-72 hours for pathogen identification and often have suboptimal sensitivity, resulting in delays in initiating targeted therapy and reliance on broad-spectrum empirical antibiotics [5,6]. Such delays can worsen outcomes in critically ill adults, where rapid progression of infection is common [7].

Recent advances in molecular diagnostics, particularly the BioFire® FilmArray® Pneumonia Panel (BFPP; BioFire Diagnostics, LLC, Salt Lake City, UT, USA), offer a promising alternative. This multiplex PCR-based assay can detect over 20 bacterial pathogens, several viruses, and fungi, along with key antimicrobial resistance markers, directly from respiratory samples within hours [8]. The rapid and comprehensive nature of BFPP enables clinicians to tailor antimicrobial therapy earlier, reducing inappropriate antibiotic exposure and supporting antimicrobial stewardship [9,10].

The integration of BFPP into diagnostic workflows has the potential to transform VAP management by improving diagnostic accuracy, shortening ICU stays, lowering healthcare costs, and ultimately improving patient survival [11]. The aim of this study was to evaluate the comparative effectiveness of BFPP and conventional culture sensitivity testing in adult ICU patients with VAP, focusing on diagnostic performance and clinical outcomes.

## Materials and methods

The study was conducted in the Himalayan Institute of Medical Sciences, Swami Rama Himalayan University, a North Indian tertiary care medical college in Dehradun, India, over a duration of 12 months. Ethical clearance was obtained from the institutional ethics committee of the institution (approval number: SRHU/HIMS/RC/2025/389), and informed written consent was secured from all participants wherever possible or their relatives. Patients who were mechanically ventilated and developed clinical features suggestive of VAP were considered for inclusion. Eligible patients were above 18 years of age and met the National Healthcare Safety Network (NHSN) criteria for infection-related ventilator-associated complications (IVAC) [12]. Patients with a known allergy or adverse reactions to antibiotics used in the study, those with a history of lung transplantation, or those receiving immunosuppressive therapy were excluded.

This was designed as an observational longitudinal study. Based on hospital data from the preceding three years, the VAP rate was estimated at 7.5%.

The sample size was calculated using the formula $n = \frac{Z_{1-\alpha/2}^{2} \times p \times (1-p)}{d^{2}}$ with p set at 0.075, d at 5%, and Z at 1.96 for a 95% confidence level. This yielded a required sample size of 106 patients. These were divided equally into two groups based on the availability of tests: Group A (n=53) underwent both BFPP and conventional culture sensitivity testing, while Group B (n=53) underwent only bacterial culture and sensitivity testing.

Respiratory samples were obtained via bronchoalveolar lavage (BAL), mini-BAL, tracheal aspirate, or tracheostomy aspirate. Group A samples were analyzed using the BFPP according to the manufacturer’s protocol, whereas Group B samples were processed using standard microbiological techniques for bacterial culture and sensitivity. Data collection was carried out using a structured case reporting form, which included demographic details, comorbidities, Acute Physiology and Chronic Health Evaluation II (APACHE II) scores [13], antibiotic therapy, and clinical outcomes.

Clinical outcomes compared between groups included duration of mechanical ventilation, length of ICU stay, total hospital stay, and mortality. Data were entered into Microsoft Excel (Microsoft Corp., Redmond, WA, USA) and analyzed using IBM SPSS Statistics software, version 26 (IBM Corp., Armonk, NY, USA). Categorical variables were expressed as frequencies and percentages, while continuous variables were summarized as mean ± standard deviation or median with interquartile range, depending on distribution. Between-group comparisons were performed using Student’s t-test for continuous variables and the chi-square or Fisher’s exact test for categorical variables. A p-value of <0.05 was considered statistically significant.

## Results

The study enrolled a total of 106 patients who fulfilled the inclusion criteria, with 53 patients in the culture group and 53 in the BFPP group. The mean age of the culture group was 63.2 ± 13.5 years, while the BFPP group had a mean age of 58.4 ± 17.1 years, with no statistically significant difference (p = 0.12). Age distribution across groups showed that 62.3% (33/53) of patients in the culture group and 69.8% (37/53) in the BFPP group were below 60 years, while 37.7% (20/53) and 30.2% (16/53), respectively, were above 60 years (p = 0.32). The sex distribution was also comparable, with 15 females and 38 males in the culture group and 16 females and 37 males in the BFPP group (p = 0.83). However, there was no significant difference in the severity of illness as reflected by APACHE II scores (Table 1).

**Table 1:** Baseline Characteristics of the Study Groups BFPP: BioFire® FilmArray® Pneumonia Panel; APACHE-II: Acute Physiology and Chronic Health Evaluation II

Variable	Culture Group (n=53)	BFPP Group (n=53)	p-value
Age (years), mean ± SD	63.2 ± 13.5	58.4 ± 17.1	0.12
Age <60 years	33 (62.3%)	37 (69.8%)	0.32
Age ≥60 years	20 (37.7%)	16 (30.2%)	0.32
Sex: Female	15 (28.3%)	16 (30.2%)	0.83
Sex: Male	38 (71.7%)	37 (69.8%)	0.83
APACHE II Score, mean ± SD	30.7 ± 6.2	29.5 ± 7.1	0.2
Comorbidities: Diabetes mellitus	21 (39.6%)	19 (35.8%)	0.68
Comorbidities: Hypertension	24 (45.2%)	20 (37.7%)	0.44
Comorbidities: Chronic obstructive pulmonary disease	10 (18.9%)	12 (22.6%)	0.64
Comorbidities: Chronic kidney disease	8 (15.1%)	6 (11.3%)	0.58
Duration of mechanical ventilation (days), median (IQR)	12 (8–18)	10 (7–15)	0.09
ICU stay (days), median (IQR)	13 (9–18)	9 (7–13)	0.021
Hospital stay (days), median (IQR)	18 (12–24)	14 (10–19)	0.03

**Figure 1:**
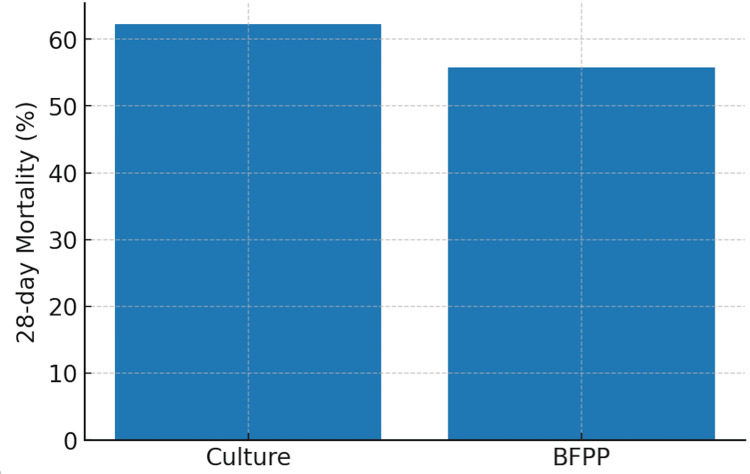
Comparison of 28-Day Mortality BFPP: BioFire® FilmArray® Pneumonia Panel

**Figure 2:**
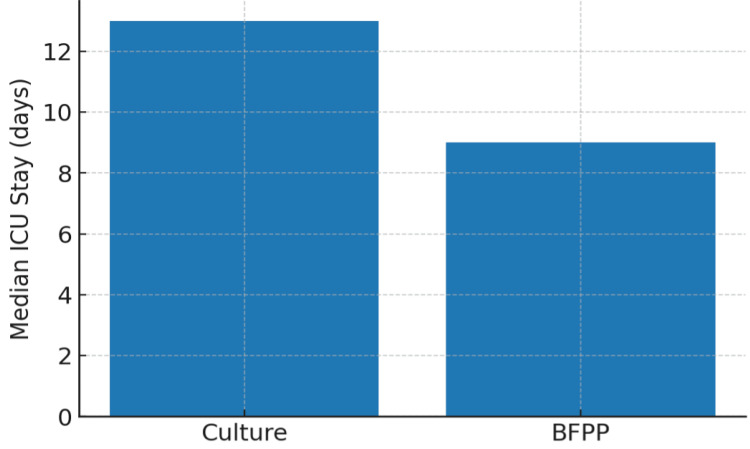
ICU Stay in Positive Cases BFPP: BioFire® FilmArray® Pneumonia Panel

In terms of outcomes, the 28-day mortality rate was 62.3% (33/53) in the culture group and 55.8% (30/53) in the BFPP group, though this difference did not reach statistical significance (p = 0.21) (Figure 1). Patients with positive results by BFPP had a significantly shorter median ICU stay of nine days compared to 13 days in the culture-positive group (p = 0.021), while no significant difference was observed in ICU stay among patients with negative results (Figure 2). Mortality analysis stratified by ventilation duration showed that patients ventilated for ≤7 days had lower mortality in the BFPP group (23.5%, 8/34) compared to the culture group (35.3%, 12/34), whereas mortality in patients ventilated for more than seven days was high in both groups (77.8%, 18/21 vs. 80.8%, 19/21). Similarly, ICU stay was consistently shorter in the BFPP group across both short-term (≤ 7 days) and prolonged (> 7 days) admissions (Table 2).

**Table 2:** Comparison of Results Between Culture and BFPP Groups BFPP: BioFire® FilmArray® Pneumonia Panel

Outcome Parameter	Culture Group (n=53)	BFPP Group (n=53)	p-value
28-day mortality	33 (62.3%)	30 (55.8%)	0.21
ICU stay: Positive cases (days, median (IQR))	13 (9–18)	9 (7–13)	0.021
ICU stay: Negative cases (days, median (IQR))	7 (5–11)	6 (4–9)	0.237
Ventilation ≤7 days: Mortality	12/34 (35.3%)	8/34 (23.5%)	0.05
ICU stay ≤7 days (median (IQR))	7 (5–11)	6 (4–9)	0.041
ICU stay >7 days (median (IQR))	13 (9–18)	9 (7–13)	–
Hospital stay (days, median (IQR))	18 (12–24)	14 (10–19)	0.03
Antibiotic duration <60 years (days, median (IQR))	10 (7–14)	9 (6–12)	0.47
Antibiotic duration ≥60 years (days, median (IQR))	12 (9–15)	11 (8–14)	0.12
Correlation ventilation: Antibiotic use (ρ)	0.61 (p=0.001)	0.54 (p=0.001)	–

**Figure 3:**
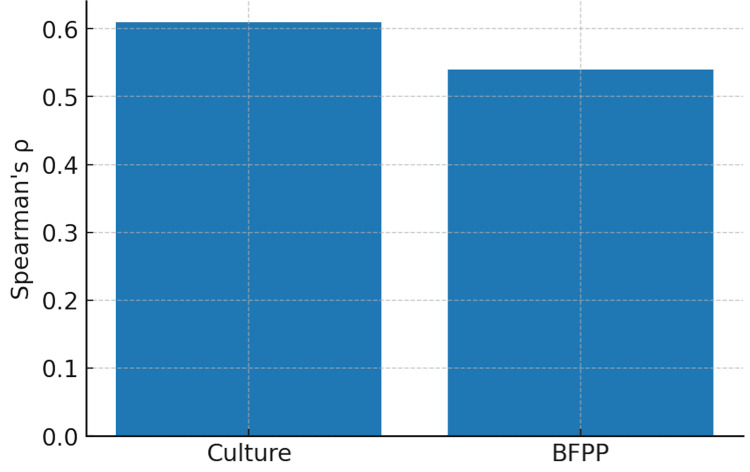
Correlation Between Ventilation and Antibiotic Use BFPP: BioFire® FilmArray® Pneumonia Panel

Correlation analyses indicated strong positive associations between hospital stay and other clinical variables in both groups, though the relationship was slightly stronger in culture patients (ρ = 0.78) compared to BFPP (ρ = 0.66). Similarly, antibiotic usage was strongly correlated with ventilation duration in both groups, but the correlation was higher in the culture arm (Figure 3).

Overall, the use of BFPP was associated with reduced ICU stay, earlier extubation, and lower mortality trends compared to conventional culture, particularly in patients with microbiologically confirmed VAP. Although some mortality differences were not statistically significant, the consistent pattern of improved outcomes in the BFPP group highlights the clinical advantage of rapid molecular diagnostics in critically ill patients. 

## Discussion

The present study was conducted to evaluate the clinical impact of the BFPP in comparison with conventional culture sensitivity testing for the diagnosis and management of VAP. Even though randomization was not done at the time of division into groups, the results indicate that while baseline characteristics such as age and sex were comparable between groups, patients in the culture group had slightly higher APACHE II scores, which was not statistically significant. Despite this difference, outcomes consistently favored patients diagnosed with BFPP, particularly in terms of reduced ICU stay, earlier extubation, and lower mortality trends, even though some differences did not achieve statistical significance. These findings highlight the potential positive association of integrating rapid molecular diagnostics into clinical practice for critically ill patients.

The mortality analysis showed that the BFPP group had a lower 28-day mortality rate (55.8%, 30/53) compared with the culture group (62.3%, 33/53), although the difference was not statistically significant. Similar patterns have been reported in previous studies. Kakati et al. observed that rapid molecular diagnostics, including the BFPP, were associated with reductions in mortality and earlier initiation of appropriate therapy, although statistical significance was sometimes lacking due to small sample sizes and variations in baseline illness severity [10]. Metlay and colleagues also demonstrated that implementation of rapid PCR-based pneumonia panels shortened the time to targeted therapy and improved survival rates in ICU patients [11]. The lack of statistical significance in the current study may be explained by the higher baseline APACHE II scores in both groups, as severity of illness is an independent predictor of mortality [13]. Nonetheless, the trend toward improved survival supports the positive association of BFPP in guiding timely therapy.

An important observation in the current study was the significant reduction in ICU stay among BFPP-positive patients. The median ICU stay in this group was nine days compared to 13 days in the culture group. This result aligns with the work of Thakur et al., who reported that BFPP allowed for faster initiation of targeted antimicrobial therapy, which translated into shorter ICU stays and improved resource utilization [8]. Mishra et al. similarly demonstrated that BFPP reduced ICU stay duration by providing results within 1.3 hours compared to an average of three days for culture-based methods [14]. Shortening the length of ICU stay has profound implications for both patient outcomes and healthcare costs, as prolonged ICU admissions are associated with increased risk of nosocomial infections, higher mortality, and greater economic burden [5]. The findings of the present study are therefore consistent with the growing body of evidence that early pathogen-directed interventions facilitated by molecular diagnostics are associated with earlier stabilization and faster recovery.

Ventilation outcomes also supported the use of BFPP. Patients ventilated for ≤7 days had a lower mortality rate in the BFPP group (23.5%, 8/34) compared to the culture group (35.3%, 12/34). For those ventilated longer than seven days, mortality remained high in both groups, reflecting the well-documented association between prolonged mechanical ventilation and adverse outcomes. Meduri and colleagues emphasized that extended ventilatory support is an independent risk factor for poor prognosis in VAP due to increased susceptibility to secondary infections and organ dysfunction [4]. Nevertheless, the trend toward better outcomes with BFPP in shorter ventilation categories suggests that early diagnosis and intervention are associated with preventing prolonged ventilation, thereby reducing mortality. This observation concurs with studies by Buchan et al. and Chen et al., who found that rapid diagnostics allowed clinicians to optimize antimicrobial regimens sooner, which in turn facilitated earlier extubation [9, 15].

Hospital stay was also significantly shorter in the BFPP group, with a median duration of 14 days compared to 18 days in the culture group. This finding supports the observations of Jain and colleagues, who emphasized that rapid diagnostics contribute to earlier discharge and reduced healthcare costs [16]. In resource-limited settings, this difference is particularly important as ICU beds are often scarce, and reducing length of stay can improve overall efficiency of healthcare delivery. Furthermore, a shorter hospital stay reduces the risk of nosocomial infections and antibiotic-related complications, thereby improving both patient safety and institutional outcomes.

The correlation between antibiotic use and ventilation was strong in both groups, though slightly higher in culture patients (ρ = 0.61 vs. 0.54). This indicates that prolonged ventilation was consistently associated with prolonged antibiotic use, a finding consistent with antimicrobial stewardship literature. The BFPP, by enabling earlier de-escalation or discontinuation of antibiotics, has been shown in previous studies to reduce unnecessary antibiotic exposure. Buchan et al. reported that the use of BFPP modified antibiotic therapy in 70.7% of patients, with de-escalation achieved in nearly half [9]. Similarly, Kakati and colleagues demonstrated that antibiotic days were significantly reduced when therapy was guided by BFPP [10]. The present study reinforces these findings, as patients in the BFPP group had shorter antibiotic durations across age categories, even though the differences were not statistically significant.

Despite its advantages, BFPP also has limitations. The current study found that mortality in patients with negative BFPP results was slightly higher compared to those with negative culture results, a difference that was statistically significant. This highlights the challenge of interpreting negative molecular results, which may occur due to pathogens not included in the panel or because residual clinical features are due to non-infectious causes. Meehan pointed out that false negatives and detection limits must be considered when using molecular diagnostics, and clinicians should interpret results in conjunction with clinical judgment and conventional methods [17]. This limitation emphasizes that BFPP should serve as an adjunct rather than a replacement for cultures, particularly as culture methods remain valuable for detecting uncommon pathogens and providing complete antimicrobial susceptibility profiles [18].

Cost considerations must also be addressed. Although BFPP provides rapid and accurate results, it is more expensive than conventional culture methods. This can limit its widespread implementation in resource-limited healthcare settings. However, several studies have demonstrated that the initial expense of molecular diagnostics is offset by savings from reduced ICU and hospital stays, decreased antibiotic usage, and improved outcomes. Ginocchio and colleagues stressed that the broader economic benefits of BFPP, including reduced antimicrobial resistance pressure, justify its integration into clinical workflows [19]. In the present study, the consistent trend of improved outcomes with BFPP supports the notion that its benefits may outweigh the costs in critical care settings.

Overall, the results of this study are consistent with those of previous investigations, which have demonstrated that rapid molecular diagnostics are associated with improvement in the timeliness of targeted therapy, reduced ICU and hospital stays, enhanced antimicrobial stewardship, and better survival trends. While not all outcomes reached statistical significance, the consistent direction of benefit across multiple parameters underscores the clinical utility of BFPP.

Limitations of the study

Small sample size, non-randomization of patients in groups, and observational design of the study limit the direct association of rapid molecular testing to improved ICU stay and reduced ventilation days. As the sampling was convenience sampling, the presence of selection bias cannot be ruled out, and this acts as one of the limitations of this study. Further, due to the small study group, subgroup analysis based on the severity of illness and comorbidities was not possible.

Future scope

Future studies with larger cohorts, stratification by illness severity, and cost-effectiveness analyses will help strengthen the evidence base for incorporating BFPP into routine VAP management.

## Conclusions

The present study demonstrates that the BFPP provides faster and more comprehensive diagnostic information than conventional culture sensitivity testing in patients with VAP. Although mortality reduction did not reach statistical significance, the use of BFPP was consistently associated with shorter ICU and hospital stays, earlier extubation, and improved antibiotic stewardship. These findings support the integration of rapid molecular diagnostics as an adjunct to conventional methods for the timely and accurate management of critically ill patients with VAP.
